# Experiments *on* Galvanic Contractions *Excited upon Warm and Principally upon Cold Blooded Animals*

**Published:** 1803-02-01

**Authors:** Martin Tupper

**Affiliations:** Exeter College, Oxford


### EXPERIMENTS *on* GALVANIC CONTRACTIONS *excited upon warm and principally upon cold blooded Animals; by* MARTIN TUPPER, *of Exeter College, Oxford.*

A Series of experiments was instituted some years ago, in consequence of contractions having been then first observed in the muscular fibres of animals when treated in a certain manner with metallic and other substances, and the results of these were arranged under the name of Galvanic phenomena. Such effects are not however produced on the muscular fibre alone, for the nerves which are distributed to the organs of sense may also be excited by the Galvanic irritation, so as to produce impressions similar to the sensations themselves.

This branch of the science of Galvanism has been so fully treated of by Galvani, Humboldt, and other philosophers, that it will not be thought necessary to enter into an extensive detail of any particular part of it. These experiments are indeed, in a great measure, a repetition of those which Humboldt has already so ably conducted; I have however diversified them in many essential respects; and although their results do not in some circumstances correspond with his observations, still I am satisfied that if I have been less successful, it can only be attributed to a lesser degree of excitability in the frogs I made use of, which were however as healthy and vigorous as any that could be procured in this country in the months of July, August, and September.

I have chiefly conducted the following experiments with zinc and silver armatures, connected to the muscles and nerves, and made to communicate either by a simple metallic arc, or by one variously complicated, and composed of different substances. The earths, various fluids, animal and vegetable matter in different states, and the living human body, have also been made use of in many instances, as well in the armatures as in the arcs.

I have chiefly made use of the hind and fore extremities of frogs, very powerful effects may however be produced upon the larger warm blooded animals by the Galvanic battery, and I have in many instances excited very strong contractions in mice, dogs, and oxen, by using a large apparatus of that kind.

I have not attempted to explain the cause of any of the following results; but it is hoped that this branch of Galvanism, which is so likely to explain some of the most important portant functions of the animal economy, will be prosecuted by Physiologists with that ardour which it so truly deserves.

Some of the experiments which will be related would have been passed over, had it not been for the sake of completing the series from the most simple to the more complicated method of exciting Galvanic contractions in animals.

Experiment 1. *Of Galvanic Effects excited in the Muscular Fibres.*

When the sciatic nerves of a frog were placed upon a plate of zinc, and the limbs to which they were organically united and distributed upon a plate of silver, and communication was produced between the two armatures by a silver wire or excitatory arc, very strong muscular contractions took place in both the limbs.

When one sciatic nerve was armed, contractions were excited only in the limb to which it was distributed. The contractions in these two experiments were equally powerful, whether the silver arc touched the muscles or their armature. The effects were similar, whether the muscles were armed with zinc, and the nerves with silver, or the contrary. When communication was produced between the armature of the nerve and the nerve itself, contractions were excited, but only in newly killed and very excitable animals. When only the posterior tibial nerve was armed, the contraction took place in the flexor muscles of the foot; when only the anterior tibial, they were excited in the extensors. When the two nerves were armed, the contractions of the flexors were in some degree counteracted by those of the extensors.

Amongst other evident inferences, it appears from these experiments, that it is in a great degree, if not altogether, immaterial whether the armature of the muscles be extended and in contact with them all, or whether it simply consist of a point, in contact with only a small part of a muscle.

Exp. 2. *Of Galvanic Effects when Ligatures are made on different Parts of the Nerves.*

When a cotton thread ligature was made upon one or both of the sciatic nerves, and the circle was completed, (taking care, at the same time, that the portion of the nerve between the ligature and the limbs did not touch the armature of the nerve behind it) the effects were positive.

tive. When a silk ligature was substituted the results were the same. When communication was produced between the armature of the nerve, and that part of it between the ligature and the muscles, strong contractions took place; and in animals possessing a high degree of excitability, if the circle was completed from the same armature to the portion of the nerve between it and the ligature, the effects were also positive. In these experiments no suspicions could be entertained that the ligatures were not sufficiently tight. The ligature remaining as before, if the portion of nerve between it and the muscles was completely surrounded by a metallic or any other exciting or conducting substance, no effects ensued when the circle was completed. When no ligature was made, and the whole nerve was surrounded in a similar manner, although the effects were not negative, yet they very soon became less and less powerful, and at last ceased altogether. When the substance which surrounded the nerve was removed, the contractions were renewed with great force. When a ligature was made at the insertion of the nerve into the muscle, no effects could be produced in the corresponding limb, but they were positive in the opposite, which is a proof that the tied nerve was capable of conducting the Galvanic influence. When the above ligature was removed, no contractions whatever could be excited in the limb to which the nerve which had been tied was distributed, until it had been dissected from between the muscles of the thigh. These curious results offer a most fruitful field for further investigation; they have been ingeniously explained, but many circumstances, I think, remain to be cleared up, before their cause can be known.

Exp. 3. *Of Galvanic Effects when the Nerves have been divided.*

When the sciatic nerves of a frog had been divided, and the separated portions armed as usual, whilst the other part of the nerves together with the limbs to which they were organically united, were also placed on an armature, and the cut ends were brought into contact and the communication produced as before, the effects were positive. When the divided portions were placed side by side, or were each brought into contact with the nerve of which they had not formed a part, positive results also ensued. When the nerves of a dog, mouse, or fish, were substituted, the effects were the same. In these experiments the animal arc consisted of nerves not united, and nerves organically united with muscles and muscles. Exp.

Exp. 4. Having armed a portion of nerve with zinc, so that it hung over the edge of the metal, I brought it into contact with the nerve from which it had been separated, and which was still united to the limb; then having placed the nerve of the opposite limb upon the united portion of the other nerve and completed the circle, strong muscular contractions were excited in both the limbs.

Exp. 5. Every thing being disposed as in the first experiment in 3, excepting that the cut portions were only brought into proximity upon a narrow blade of glass interposed between the armatures, when the circle was completed the effects were positive. Wishing to ascertain whether these contractions depended upon the conducting power of moisture which might be interposed between the cut ends of the nerves, I tolerably accurately dried the intervening space and found that slight contractions might still be produced, but that when all moisture was excluded they did not take place, although the cut ends were brought as near to one another as possible.

I fear that some cause prevented my success in these experiments, for Humboldt has obtained positive results in trials of a similar kind. He says, that a nerve very often propagates the Galvanic influence to another at some distance from it, when very excitable and newly killed animals are employed. I have sometimes observed that muscular contractions have taken place under these circumstances, but I cannot determine whether they arose from any influence propagated as an aura from one cut end of the nerve to the other. These effects generally cease very soon, and they may often be observed when no excitatory arc whatever is employed, and even when the limbs are placed upon a table, without the contact of any apparent stimulating cause.

Exp. 6. *Of Galvanic Effects, the Nerves being neither divided nor tied.*

The sciatic nerves and limbs of a frog being disposed as before, a piece of cold boiled muscle was placed upon the armature of the former; and when the circle was completed between the boiled muscle and the armature of the latter, the effects were positive. It appeared to be immaterial whether the nerve was placed upon the boiled muscle, and the arc was brought into contact with the two armatures, or whether the boiled muscle was placed upon the limbs, and communication was produced between it and the armature of the nerves.

 The

The sciatic nerves and inferior extremities of a very lively frog having been placed on their respective armatures, I constituted an excitatory arc of the following substances, and disposed them in the order of zinc, roasted muscle, silver, spawn of frog, zinc, raw liver, silver, intestine, zinc, boiled beef, steel, and the circle being completed between the last piece of zinc in the arc and the (silver) armature of the limbs, the effects were much stronger than when the arc communicated with the boiled beef and silver; but when the steel and the silver armature were made the points of contact with the arc, the contractions were very powerful, although the animals had been killed and skinned nearly two hours.

In the above, as well as in all other similar experiments, I have invariably found, that the effects are always more powerful when the armature of the muscles or nerves is brought into direct contact with a metallic substance in the compound excitatory arc, than when a muscular or a fluid body is interposed.

Exp. 7. Having then excited Galvanic contractions by composing the arc of various heterogeneous substances, I next placed myself in the circle of communication, and obtained positive results, when I touched the armature of the nerves with a bar of steel held in my left hand, and that of the muscles with a piece of fresh muscle held in my right. The effects were the same when a column of blood, water, urine, or milk was substituted for the piece of muscle.

Exp. 8. When the sciatic nerves and lower limbs of a frog had been armed as usual, I moistened the finger of my left hand, and touched the (zinc) armature of the former, and with a silver wire, held in my right, I touched the (silver) armature of the latter, but no contractions were excited; when a bar of zinc was substituted for the silver wire, they became very powerful. When a finger of the right hand touched the armature of the muscles, and the (zinc) armature of the nerves was rubbed with a bar of zinc, the effects were again negative; but the instant silver was made use of they became positive. These and similar experiments were often repeated with similar results.

I have seldom succeeded in producing positive effects, when the excitatory arc consisted of the (zinc) armature of the nerve, a finger of my left hand, my body, and iron wire, and more particularly silver or brass wire, and the (silver) armature of the muscles; but contractions took place, place, when either of the above three wires were placed in contact with the armature of the nerve, and a finger of the right hand touched the armature of the muscles.

Exp. 9. In the following experiment, in which the armature of the nerves was in contact with a bar of steel held in my left hand, my right joined with the left of a gentleman, his right with the left of another, and so on to a seventh, who completed the circle, by touching the armature of the muscles with a silver wire, the contractions were as strong as if the excitatory arc had been composed of only steel, one person, and silver. If silk, wax, glass, or amber, interrupted the arc, all effects ceased; but when any of the metals, boiled or raw muscle, water, urine, alcohol, the ethers, or the acids, formed a link of the chain, the contractions again assumed their former strength.

Exp. 10. When a quantity of raw muscle was placed upon the (zinc) armature of the nerves, upon this a plate of gold, and upon this again another portion of raw muscle; and raw muscle, a silver plate, and raw muscle, were disposed upon the other (silver) armature, when the circle was completed by producing communication between the muscle on the gold and the muscle on the silver with a silver, brass, steel, or iron wire, the effects were negative. When a plate of silver was placed upon the last piece of muscle on the silver side, and the communication was established, the effects were positive; but if the same silver plate was placed on the zinc side, the effects again became negative. When zinc was placed upon the muscle on either side, and the circle was completed, the contractions were in both instances very powerful.

In other similar experiments, I placed raw muscle on the zinc, and zinc again on the muscle, and when the circle was completed, the effects were positive; but when zinc was placed upon zinc and the muscle upon this, and the communication was then established, only very slight effects were excited. This experiment was repeated several times with the same result. It appears to be a curious circumstance, that the first method should present a positive, and the latter a negative result.

Exp. 11. Galvanic contractions are equally powerful when they are excited by a long as by a short circuit. I have been successful when the arc consisted of a bar of steel four inches long, a bar of silver of the same length, two men, and 18 feet of iron wire. Another experiment, which afforded instantaneous and positive results, was made in the open air with an excitatory arc of iron wire, about 1870 feet in length. It was immaterial whether the wire was supported by poles, or whether it was in contact with the ground, which was very wet from a late rain. It appears from this, that water is not (at least) a better, and probably a worse, conductor than metal; for as the whole length of the wire was not in contact with the ground, if water had been the best conductor, positive effects would not have resulted, because the Galvanic influence would probably have been diffused over the moist soil before it could have completed the circle.

Exp. 12. It was next wished to be ascertained, whether muscular contractions could be produced by forming the circuit from the armature of nerves to the armature of parts to which they were not distributed: With this view I dissected a frog, so that the sciatic nerves remained united to the upper half of the body, and the communication being effected, moderately strong contractions were produced in the upper limbs, which could not however be renewed twenty minutes after the death of the animal. This experiment was repeated several times, but seldom with much success; muscular contractions were however always very strong in the muscles at the posterior part of the chest and abdomen, as high up as ¼ of an inch from the origin of the sciatic nerves. I learnt from subsequent trials, that in experiments of this kind, the contractions were produced in consequence of the sciatic, lumbal, and intercostal nerves forming a chain of communication from the armature of the former to the axillary nerves; for after having partially separated the intercostals from the surrounding parts, and placed the last upon the sciatics, the last but one upon the last, and so on, until a chain of several nerves was formed, the contractions became very strong in those muscles to which any of the above nerves were distributed, when the circle was completed; but when the chain was interrupted, by removing the first intercostal from the contact of the sciatic, the effects again became negative. I can assign no reason why the spinal marrow of the animal did not conduct the Galvanic influence from the sciatic to the axillary nerves, as well as the medium of the lumbals and intercostals, more particularly as, that when a nerve has been entirely separated from seems the body, and is made a constituent part of the chain, it seems to be of no consequence whether it is placed in a situation corresponding with the supposed motion of the nervous influence in it, or in a contrary one. Many apparently contradictory results, however, take place in experiments of this nature. It would seem, that the effects are positive or negative, in some degree, as those nerves which are placed in a situation opposed to the motion of the fluid in them, are connected to the surrounding muscles by the cellular membrane or not.

Exp. 13. It is not necessary, to produce Galvanic effects, that any portion of the nerves should be separated from the contiguous parts. Very strong contractions were produced in an instance of this kind, after the animal had been killed and skinned about nineteen hours. Thus, also, if a piece of muscle be separated from any part of the body, (probably excepting the heart) and both ends are armed and the circle completed, it will contract very strongly. These experiments again, in some measure, appear to be contradictory to those related under Exp. 2, in which the effects were stated to be negative when the nerves had been separated from the connecting parts, and were surrounded by a muscular or any other conducting substance.

*New Burlington Street, Jan. 9,* 1803.

